# Skill-Based Electronic Gaming Machines: Features that Mimic Video Gaming, Features that could Contribute to Harm, and Their Potential Attraction to Different Groups

**DOI:** 10.1007/s10899-024-10296-5

**Published:** 2024-04-23

**Authors:** Philip Newall, Matthew Rockloff, Hannah Thorne, Alex M. T. Russell, Tess Visintin, Nerilee Hing, Matthew Browne, Georgia Dellosa

**Affiliations:** 1 Experimental Gambling Research Laboratory, School of Health, Medical and Applied Sciences, CQUniversity, 6 University Dr, CQUniversity, Sydney, QLD 4670 Australia; 2 School of Psychological Science, University of Bristol, Bristol, UK; 3 Experimental Gambling Research Laboratory, School of Human, Medical, and Applied Sciences, CQUniversity, 44 Greenhill Rd, Wayville, SA 5034 Australia

**Keywords:** Electronic gaming machines, EGMs, Gambling product design, Structural characteristics

## Abstract

New gambling products have been developed over time as technology permits. For example, early mechanical slot machines were later replaced by electronic gaming machines (EGMs), which enabled a faster speed of play and more immersive experience. EGMs have in the decades since their invention become one of the main drivers of gambling expenditure worldwide and are one of the gambling products most strongly associated with harm. This literature review considers research relevant to a new subcategory of EGM, ‘skill-based’ EGMs, termed ‘SGMs’ here. SGMs can be highly varied in content, with some representing a minimal departure from EGMs, where the typical bonus round is replaced by some skill-based activity, such as a simple video game, which could increase the machine’s appeal. Other SGMs feature more radical departures from conventional EGMs, such as multiplayer games using intellectual property from popular TV shows or video games. These skill-based elements could tap into common gambling fallacies such as the illusion of control, and therefore facilitate harmful engagement. SGMs could also be less harmful than current EGMs, if skill-based elements break the dissociative states associated with EGM gambling. The intellectual property used in SGMs may increase their appeal among people who generally do not gamble, and the skill-based elements could increase their interest among gamblers who predominately prefer skill-based gambling formats such as sports betting. The novelty and varied content of SGMs present many open questions, which research should aim to address in future.

## Introduction

New gambling products are always being developed. The present paper reviews research on a new class of electronic gaming machine (EGM) which incorporates aspects of skill-based play, defined here as skill-based electronic gaming machines (SGMs). SGMs are one of the most recent innovations in EGM design, having been, at the time of writing, licensed for use in only a few jurisdictions including a handful of US states. These states include Connecticut, New Mexico, Nevada, New Jersey, and Oklahoma (Hoskins & Hoskins, 2020; Ofgang, 2017; Pickering et al., 2020), with Nevada and New Jersey being the earliest to grant approvals in 2015 (Larche et al., 2016; Legato, 2021). In Australia, trials and regulatory sandboxes—machines closely monitored by regulatory authorities in specific locations— are being used to monitor skill-based gambling machines. At the time of writing this paper, one SGM had been approved for use in New South Wales, Victoria, and Western Australia (Pop-shots - Witches Coven), while four games had been approved for use in Queensland (Pop-shots - Witches Coven Deluxe, Pop Shots - Wild Mermaid Deluxe, Megamatch Lucky Harvest and Megamatch Jelly Kingdom) (personal communication, Australian gambling regulators).

As will be described later, SGMs can be very broad in scope, but one of the key mechanics is to include aspects from video games within the electronic gaming machine. Like many aspects of life, performance on a video game is something which involves an element of experience or skill. Importantly, performance on an SGM’s skill-based features, whether video game-based or otherwise, are designed to in some way impact the gambling outcomes or experience produced by the machine.

Literature relevant to skill-based gaming machines (SGMs) was investigated and subjected to a narrative review (Ferrari, 2015). This was done because SGMs are a relatively modern gambling product that are currently only deployed in a few jurisdictions. These facts meant that the alternative of a systematic review, based around key search terms placed into scientific databases, was unlikely to yield any direct hits beyond those already covered in a simple search. Furthermore, grey literature on the topic is likely to be limited and difficult to capture. The review also more broadly covers a range of gambling literature on international developments in gambling product design, and the extent to which these innovations are relevant to gambler skill or the illusion of control when no skill element is present. A narrative review was chosen as being the most appropriate way to answer this research question, since these broader issues are clearly relevant to a contemporary understanding of SGMs.

## Background

The purpose of this section is to present a contextual overview of relevant areas from gambling research for the literature review. The design of gambling machines evolves with technology. The first gambling machines were designed in the late 19th century and were based on three mechanical spinning reels and a single “payline”, where two or more matching symbols produced a payout to the gambler (Livingstone, 2017). These first machines were popular with gamblers and cheap for gambling operators to maintain and operate. Nevertheless, there have always been significant incentives for developers to innovate their design to draw in new generations of gamblers. One significant shift was the move from mechanical to electronic gaming machines (EGMs) (Livingstone, 2017). EGMs allow for a faster speed of play and more immersive experience, providing a greater array of betting options and have more exciting audio-visual effects than mechanical machines did (Schüll, 2012). The introduction of EGMs facilitated the widespread introduction of multi-line betting as well as multi-way betting (e.g., ReelPower machines where players bet on columns rather than lines, and thus all lines are available for potential wins) but also the enhanced potential for “losses disguised as wins”, where a small payout is received that is less than the size of the original bet (Dixon et al., 2010).

However, SGMs are not the first ever gambling machine to introduce some skill-based aspect. The oldest type of gambling machine in the UK is known as a “fruit” machine, which the UK government would not license for use in pubs if they were based purely on chance. For this reason, fruit machines have “nudge” and “hold” buttons, which are intended to create some level of skill to sidestep this regulation (TVC Leisure, 2016). However, these features are largely symbolic, as their skilful use still does not enable any gamblers to have positive long-run chances of winning. Furthermore, they are not the only example of UK gambling machine developers finding creative responses to government regulations. For example, “fixed-odds betting terminals” are only allowed in bookmaker shops on UK high streets because the random events that determine the payoffs to gamblers occur at the bookmaker’s headquarters, and not on the premises of the shop itself (Cassidy, 2020). Similarly, in Canada video lottery terminals (VLTs) mimic the operation of EGMs but each play is instead a purchase of an electronic lottery ticket from a fixed pool that is maintained off-site. However, as will be seen later in this review, SGMs can form a much more radical alteration of the typical EGM’s structural characteristics than these examples from Australia, the UK and Canada.

One traditional demarcation in gambling is between *unskilled* and *skilled* gambling products. EGMs are one of the key unskilled gambling products of interest due to their international popularity (The Economist, 2017) and strong association compared to other gambling products with disordered gambling (Delfabbro et al., 2020a; Productivity Commission, 2010). EGMs are unskilled in that there is nothing that the gambler can do to affect her long-run chances of winning. The probability of each payoff happening is determined by a random draw from the EGM’s paytable, which the gambler is unable to affect (Harrigan & Dixon, 2009), and which is designed so that the gambler will lose on average (Woolley et al., 2013). This is true no matter the complexity of the EGM, with current EGMs having a number of randomly-determined features, such as bonus rounds involving free spins, which are also determined by the paytable (Rockloff et al., 2020). On relatively modern “multiline” EGMs gamblers can choose their number of betting “lines”, but this only affects the volatility of returns and not the long-run chances of winning (Harrigan et al., 2014).

In contrast, poker and sports/horse betting are two examples of skilled gambling games where different individual bets or overall betting strategies can affect a gambler’s odds of winning. This ability to affect outcomes is naturally attractive for highly skilled gamblers, a small set of whom can expect to have positive long-run chances of winning in these skilled gambling games (Kaunitz et al., 2017; Potter van Loon, van den Assem, & van Dolder, 2015). Skilled gambling games can be especially costly for less-skilled gamblers, by corollary, due to losses being funnelled to both skilful gamblers and to the gambling provider’s charge (e.g., the casino’s rake in poker, or a bookmaker’s overround in sports betting; Turner & Fritz, 2001). But skilled gambling games can still be enjoyable for people to play irrespective of skill level, as these long-run trends can take extremely long periods of play to realise returns that fall in line with skill levels (Browne, Rockloff, Blaszcynski, Allcock, & Windross, 2015). SGMs are perhaps unique in their ability to blur this demarcation between unskilled and skilled gambling products, making it difficult for gamblers to understand if their skills are leading to better returns.

One issue to touch on briefly is non-skilled hybrid electronic gambling machines where the videogame component is confined to a mini bonus-game within a purely random EGM. An example of this type of hybrid machine is “Bloomtopia” by Chill Gaming. Bloomtopia is a regular EGM with a side feature or bonus game. Along with winning points, the player can also win ‘water’, ‘sunshine’ and ‘seeds’ which are a resource the player can use to improve their ‘garden’ (a graphic of a garden to the side of the EGM reels). There is no skill element to growing the garden, and the garden side game has no impact on the outcome of the EGM or the money the player can win or cash out. There is potential that these games may hold an added attraction for certain people as there are elements that superficially resemble video or mobile phone games (such as Farmville). However, including these types of games in the current review would create confusion as these types of games cannot easily be compared to games that clearly suggest the need for skill. Hence, these types of games have not been included in this present review.

The ability of SGMs to blur the boundary between skilled and unskilled gambling products, and the appearance of the potential for positive long-term returns in SGMs, can be illustrated via the machine that might be the first ever SGM brought to market. In 2009 the EGM developer International Game Technology introduced an electronic gambling game called “Texas Hold’em Heads Up Poker” (International Game Technology, 2009). This was not a reel-based game (i.e., not a slot or pokie), and instead saw the gambler face off in an actual poker game against a computer opponent (Newall, 2023). This SGM relied on the fact that computers had already reached a level of skill in that poker format similar to that of the best poker professionals (Newall, 2018) with the documentation of a “perfect” computer player in that poker format announced in the journal *Science* only a few years later (Bowling et al., 2015). As the computer poker player in that SGM did not play perfectly, however, any imperfections in its play could hypothetically be exploited by a skilful poker professional to produce positive long-run chances of winning. However, given the high standard of computer play in that poker game, the EGM developer thought it was more likely that the SGM could instead profit from much larger potential imperfections amongst its large and diverse body of players (Christenson, 2010). Marketing material for the poker SGM explained its appeal as follows:

“This intriguing and ground-breaking game is sure to attract and entertain all types of gaming enthusiasts… All kinds of players will ante up for this new brand of poker game play.” (International Game Technology, 2009), p.15.

## Features of SGMs that Seek to Mimic Video Gaming

Research has demonstrated a number of ways in which gambling and the video gaming industry have increasingly converged (Kim & King, 2020; Kolandai-Matchett & Abbott, 2021). In many instances this convergence has involved video games becoming more like gambling, a trend that has been called “gamblification” (Macey & Hamari, 2022). “Loot boxes”, which consist of randomised in-game items that must be purchased using real money are one example of this, and which meet several defining characteristics of gambling in that they cost money and can oftentimes produce items worth less than the purchase price (Drummond et al., 2020). “Social casino games” are another example and involve the playing of traditional casino games such as slot machines purely for points. These points, despite having purely symbolic value, can however also be purchased with real money (Kim et al., 2017). Many SGMs, however, represent a similar trend acting in reverse. They use skill-based video game technology to effectuate gambling rather than simulating gambling games in purely digital form.

Many SGMs mimic video games for their skill-based content. Some SGMs involve first-person shooters video games like Doom or Half-Life. Other SGMs involve third-person fantasy games and role-playing games like World of Warcraft. Other SGMs involve direct replicas of retro video games, such as Pac-Man—games which are enduringly popular with video gamers of all ages (Pickering et al., 2020). However, as will be illustrated throughout the remainder of this review, this content varies greatly with respect to its centrality and sophistication. One SGM which has been approved for use in the Australian states of New South Wales, Western Australia and Queensland, “Pop Shots Witches Coven”, lies at one end of this spectrum. This game is mainly a multiline slots-based game, while the bonus round determining the user’s number of free spins is related to video gaming. In this bonus round, the user must pop bubbles via the machine’s touchscreen interface. This videogame feature is relatively simple, and it is relatively easy to obtain the high score on it. These features mean that few gamblers are likely to find this game particularly engrossing, or to think that their ability at the game will produce positive long-run chances of winning for them at the SGM.

Other SGMs can keep this main focus on being a multiline slots-based game but have more engrossing video games for their bonus round content (Hoskins & Hoskins, 2020). One example is the retro video game of Space Invaders, which was a renowned arcade game (Pickering et al., 2020). An SGM with this bonus round might be more attractive to gamblers than the example from the previous paragraph for two reasons. First, the game involves a higher level of complexity and is harder to obtain maximum performance and a resulting high score on it. Second, the game was very popular in the past, and so many older people may wish to play it for nostalgic reasons. This simple formula of placing various games within the bonus round of a multiline slots-based game can potentially appeal to many different users, with for example a first-person shooter game being one game which might appeal to a different, and potentially younger demographic (Pickering et al., 2020). This is a topic which will be returned to later in this review. However, for now it is sufficient to state that these games, in addition to being more engaging, may also be more likely to make gamblers think that they have positive long-run chances of winning than simpler low-skilled games like Pop Shots Witches Coven.

Other SGMs can be innovative by incorporating multiplayer features. Many of the most popular video games are multiplayer, involving several players who actively compete against one another to test their relative skills. Multiplayer games can be both retro or modern, and therefore of potential appeal to diverse gamers. Importantly, the aspect of relative skill can potentially make multiplayer games of greater interest than single player games, as a game between several highly skilled players can have a competitive appeal. Presumably, this competitive instinct also underlies the appeal of esports. One multiplayer SGM, for example, is based on the retro videogame Pac-Man and involves up to four players competing to be the last Pac-Man alive who will also win the cash prize (Pickering et al., 2020). This game is an even greater deviation from traditional EGMs, as it does not involve any traditional slots-based play. More aspects of SGM content which mimics video gaming will be discussed in later sections, in cases where they are directly relevant to the remaining topics of this review.

## Features of SGMs that could Contribute to Harmful Engagement

Compared to other gambling products, EGMs have a strong association with disordered gambling (Delfabbro et al., 2020b). Many researchers believe that this association is strengthened by numerous design features of EGMs, such as their ability to promote long gambling sessions of continuous play, and the presence of illusory payoffs such as near-misses and losses disguised as wins (Schottler Consulting, 2019). Given that these illusions are most prevalent in multiline slots-based games, it is likely that similar SGMs such as Pop Shots Witches Coven will raise similar issues. However, the unique design features of SGMs may also contribute to other aspects of harmful engagement.

An important issue for SGMs is the potential for their skill-based content to create *false* impressions of the potential for positive returns amongst some gamblers. This might especially be an issue for people who are suffering from disordered gambling, given the number of illusions about chance and skill in gambling present in this cohort (Raylu & Oei, 2004). One illusion that might be particularly relevant to SGMs is the “illusion of control”, whereby gamblers misperceive the extent to which elements of choice, mastery, or skill will boost their chances of winning (Clark & Wohl, 2021). The illusion of control is thought to be especially relevant to skilled gambling games, given the various betting strategies that can be chosen that can have some impact on the gambler’s chances of winning. However, although some gamblers can marginally improve their outcomes in skilled gambling games, most gamblers will lose money; and losing gamblers can overestimate their level of skill and ability to win in the long run. This illusion is particularly relevant in sports betting, where many gamblers think that their knowledge about the sport provides them with an opportunity to win. However, that knowledge needs to be better than the market’s knowledge implied by the current betting odds, inclusive of the provider’s overrounds, which is a high standard to beat. Previous studies have shown that expertise about a sport does not necessarily confer better predictive ability of eventual outcomes (Andersson et al., 2005). An illusion of control can lead to sports bettors selecting predictably bad bets. Sports bettors with higher levels of illusion of control, for instance, are more likely to customise their own complex bets on several constituent events (Newall et al., 2020a), which are very likely to lose in the long-run due to the high bookmaker profit margin on such long-odds bets (Newall et al., 2020a, b).

Given the unique potential for SGMs relative to traditional EGMs to enhance illusions of control amongst people who are already gambling at high levels, it is important to consider previous conceptual frameworks on elements of gambling product design (Armstrong et al., 2017; Livingstone et al., 2008; Schüll, 2012). In particular, the “VICES” framework is instructive, as it was initially developed for automated versions of traditional casino games (Armstrong et al., 2017). The dimensions in this framework correspond to “visual and auditory features”, “illusion of control”, “cognitive complexity”, “expedited play”, and “social aspects”. The relevance of these factors to SGMs, either present or potential, is discussed next.

Traditional EGMs have many audio and visual features, such as congratulatory graphics or sounds that accompany winning bets. It has been shown, for example, that changing the type of sound played to losses disguised as wins bets featuring a mixture of a gain and a larger loss can help gamblers to better understand the overall loss occurring from these bets (Dixon, Collins, Harrigan, Graydon, & Fugelsang, 2015). However, some SGMs use modern technology to create a wholly more immersive visual and audio experience. For example, the “Virtual Reality Cube” uses virtual reality technology to embed the gambler in an immersive game equal to the latest in virtual reality video game technology (Gaudiosi, 2016). The cube also has powerful subwoofer speakers underneath where the gambler stands, making this also an unusual and enhanced audio experience for a gambling game. The cube can even fill with smoke after certain in-game events. All these features are likely expensive for the EGM developer, which may be difficult for the developer to recoup based purely off the gambler’s losses. Instead, this game might be developed more to create a spectacle to entice more people to the casino floor, like the spectator element in sports and esports.

Traditional EGMs arguably have less scope to leverage illusions of control in comparison to traditional skilled gambling games such as sports betting. As discussed earlier, the most unique aspect of SGMs relative to EGMs may well be their ability to create enhanced illusions of control. This can potentially be leveraged by many SGMs in different ways. For example, a multiplayer SGM called “Deal or No Deal Poker Special” has multiple choice aspects (Ruddock, 2018). First, gamblers choose a briefcase in a manner like the TV franchise Deal or No Deal that the SGM is loosely based on. This is a purely chance-based aspect of choice, but nevertheless give an illusion of a consequential choice. Subsequently, gamblers compete with one another to “grab” playing cards to make the best five card poker hand out of the gamblers at the game. This is an aspect of play that does involve some genuine elements of decision making and coordination skill. Just like in sports betting, however, any aspect of potential genuine skill could still be overestimated by gamblers. Last, the gambler with the winning poker hand gets to either win a sure amount of money from their briefcase or open it to win a random amount (again, similar to the choice in the TV franchise). This game involves various aspects of control, some of which either do or do not influence the gambler’s long-run chances of winning.

Traditional EGMs can also be relatively low on cognitive complexity, with the only real choice in multiline slot-based games being the choice of the number of betting lines and bet size (Harrigan et al., 2014). This lack of cognitive complexity may be one reason why some traditional EGM gamblers can end up losing track of time in a dissociative state (Murch & Clark, 2021). Although SGMs will undoubtably vary on this dimension, they do have potential for a higher ceiling of cognitive complexity, which is defined here as the perceived potential for making strategic choices. For example, the multiplayer Pac-Man game, and also some example SGMs given in the next subsection based on word puzzles and fairground-type games, all present the player with complex and varied tasks in comparison to traditional EGMs. However, the true level of cognitive complexity (i.e., real rather than perceived) is likely to be less than what is found in genuine skilled gambling games, such as poker and sports/horse betting (Kaunitz et al., 2017; Potter van Loon et al., 2015).

The fast and continuous speed of play is one of the main reasons why traditional EGMs are thought to have such a strong association with disordered gambling (Productivity Commission, 2010) given the impulsivity commonly found amongst people who suffer from disordered gambling (Browne et al., 2019; Ioannidis et al., 2019). SGMs may show considerable variation on this dimension. For example, word puzzle games could present a much lower number of bets per hour than a traditional EGM, due to the extended break in play from playing the puzzle. But other SGMs could plausibly create faster speeds of play than traditional EGMs. For example, the SGM “Tempest” recreates a classic shooter video game from the Atari console (Next Gaming, n.d). In this game every shot acts as a bet, potentially allowing the gambler to place many bets in a short space of time without giving them much thought. Paradoxically, this is a gambling machine where skill could even hurt the gambler. A greater skill at the shooting aspect will increase the effective speed of play and therefore increase the gambler’s theoretical losses for any given bet size. This sort of SGM might be particularly conducive to creating a dissociative state, where a gambler loses track of the amount of money bet and time spent gambling.

Traditional EGMs are also largely solitary, with perhaps the only counterexample being progressive jackpot EGMs, which can see some competition between gamblers on different machines to try and win a jackpot (Li et al., 2016). On the one hand, solitary gambling can be especially risky (Bristow et al., 2018). On the other hand, however, being in a group of gamblers can help normalise risk-taking behaviour and having friends who gamble is a risk factor for disordered gambling (Browne et al., 2019). Additionally, gambling games involving several participants, such as poker, can potentially involve more breaks in play than solitary games such as traditional EGMs. Social aspects on gambling are therefore multifaceted, as are the social elements of SGMs. This is because SGMs have been designed that are both single player and multiplayer. The social elements of SGMs arguably vary even within these two categories, with – for example – the single player Virtual Reality Cube being designed to create a public spectacle (Gaudiosi, 2016), whereas the Tempest SGM that is based off a retro Atari game is likely to hold interest only for the gambler engaged with it. The social aspect of SGMs may well evolve over time if one of these two main types of SGMs ends up being more popular with gamblers.

In summary, the content of SGMs is sufficiently varied to create a large potential variation in terms of how their design features may contribute to harmful engagement. However, SGMs based on multiline slots-based games that contain some additional element of immersive skill-based play may be at least as harmful as traditional EGMs, which are considered to be one of the most harmful gambling products (Schottler Consulting, 2019). It is harder to make this prediction about other SGMs with more unique content. However, there are numerous aspects of individual SGMs of this type that could be uniquely harmful in comparison to EGMs, in particular the potential for their skill-based content to enhance illusions of control. It is hard to predict what potentially countervailing effects may occur from other differences from EGMs, such as social aspects or breaks in play.

## Will SGMs Attract a New Group of People Who Generally do not Gamble?

The video games market is now larger than the film and music industries combined (BBC, 2019), meaning that the SGMs containing intellectual property from popular video games such as Doom, Pac-Man, or Space Invaders may be effective at attracting people to SGMs who do not generally gamble (Pickering et al., 2020). Yet other SGMs involve games such as word puzzles, which were first played commonly as board games such as Scrabble, but which are now also played by many gamers on mobile devices. Finally, some other SGMs may reflect more basic fairground-type games, such as where a player must shoot balls into a hoop and can win prizes for good performance (Pickering et al., 2020). SGM content as varied as these examples are why this review has highlighted how hard it will be to predict their eventual popularity, and the extent to which they may become associated with disordered gambling and gambling-related harm. From this summary of previous literature, it is possible that SGM developers are going through a trial stage of developing many different types of games, with the intention to innovate on the most popular SGMs through subsequent generations of development. It certainly appears to be the case that the non-bonus slot rounds of a majority of current SGMs, or reel-based play, reflect the many years that have gone into traditional EGM development; iterating on popular themes and pay schedules.

If SGMs primarily attract non-gamblers then this appeal, at least initially, presents a lower risk of immediate harm than the other case of SGMs attracting disordered gamblers (Browne et al., 2016). However, any increase of gambling consumption can potentially lead to distal increases in harm (Grun & McKeigue, 2000; Hansen & Rossow, 2008), in particular since some gamblers can rapidly transition from low to high levels of gambling expenditure (Muggleton et al., 2021). Migrants, for example, can experience especially high rates of disordered gambling after moving to countries with large commercial gambling sectors such as Australia or the UK, if moving from a country with greater restrictions on gambling (Wardle et al., 2019). SGMs could potentially be especially attractive to non-gambling groups such as these due to their unique combination of content.

## Which Current Gamblers will Find SGMs Appealing?

While SGMs based on traditional skilled gambling games such as poker do not appear to have taken off in popularity in the way that EGM developers had hoped, the example given earlier of arguably the first SGM from 2009, “Texas Hold’em Heads Up Poker” (Newall, 2023), does demonstrate the critical issue of which demographics SGMs are marketed towards (International Game Technology, 2009). Younger gamblers and male gamblers have above-average rates of disordered gambling (Browne et al., 2019) and hence can be strong drivers of gambling revenue. Compared to other gamblers, younger gamblers and male gamblers are more likely to prefer skilled gambling products such as poker (Shead et al., 2008) and sports/horse betting (Andronicos et al., 2015). This can help explain the rationale for International Game Technology’s poker SGM, even though that product did not appear to be ultimately successful: to attract a potentially high-spending demographic to a gambling format that they show comparatively less interest in. This may be why the game’s marketing material claimed that “all kinds of players” will be interested in the game. Gambling industry reports have relatedly suggested that SGMs are popular with younger gamblers when compared to traditional EGMs (Toscano, 2018).

An important open question for research is finding out which gamblers are especially interested in SGMs, beyond the intentional skew toward younger ages (Toscano, 2018). Perhaps the most important demographic to understand is whether disordered gamblers and other people experiencing gambling-related harm are especially engaged in using SGMs. This is an issue that has been of recent interest to regulators in the UK around the topic of gambling advertising (House of Lords, 2020). Specifically, it is considered dangerous to allow gambling operators to use marketing approaches which induce higher levels of gambling amongst gamblers already experiencing gambling-related harm. An early review of gambling advertising suggested that there was no evidence that advertising induced disordered to spend more, and that gambling advertising might only shift a gambler’s consumption from one operator to another and therefore not increase the overall amount of gambling consumption, including amongst disordered gamblers (Binde, 2014). However, a later review of the literature concluded that the existing evidence, which mainly focused on gambling advertising content and gamblers’ perceptions of advertising, was not strong enough to show conclusive evidence (Newall et al., 2019). Some more recent evidence using an operator dataset of gamblers’ behaviour has shown that gambling marketing inducements can prompt increases in gambling expenditure, especially amongst gamblers with higher rates of disordered gambling symptomology (Balem et al., 2021). Evidence such as this can help support the recent greater restrictions placed around gambling marketing in countries such as Italy and Spain (Newall & Xiao, 2021).

Even if disordered gamblers do end up having a disproportionate attraction to SGMs, then it need not be the case that SGMs will necessarily drive increases in gambling-related harm. SGMs can be slower and therefore disordered gamblers may place fewer bets in each session. Additionally, if SGMs serve to mainly displace existing gambling consumption amongst disordered gamblers, and are less harmful than the other gambling products that this group are already engaging in, then SGMs may help to lower gambling-related harm amongst this group. The main way that this mechanism could hold is if SGMs turn out to be less harmful than traditional EGMs. This could occur if the bonus round content of SGMs is less harmful than the bonus rounds common on EGMs. For example, an SGM’s bonus round could act as a natural “break in play” (Blaszczynski et al., 2016), allowing gamblers an opportunity to reassess their gambling and potentially quit. This potential for a break-in-play could be the case, as traditional EGMs can for some gamblers lead to a dissociative state where the gambler loses a sense of time passing as they make many bets in quick succession (Murch & Clark, 2021). However, this assumes that the SGM’s bonus round does act to reduce a dissociative state. Video game players can also lose track of time during long periods of play (Petry et al., 2014), meaning that a video game-based bonus round may not necessarily create a natural break in play. Finally, it could also be that SGMs have differential effects depending on the gambler’s level of engagement with both the traditional EGM content and the skill-based bonus round, being more harmful than traditional EGMs for some gamblers, but being less harmful for other gamblers.

Due to their novelty and restriction to only a few jurisdictions, there has not been much previous empirical research to answer the question of which gamblers will find SGMs most appealing. A qualitative study suggests that gamblers, at least upon first experiencing an SGM, are likely to show a fair degree of confusion and struggle to fully understand these products (Gainsbury et al., 2020). While any individual’s reaction to a given SGM is likely to be highly dependent on the content of the SGM, which can vary considerably, some gamblers might be attracted to these products, and other gamblers much less so. However, this confusion around SGMs and the level of skill involved, does suggest there may be pathways for disordered gamblers to substantially overrate their chances of winning via the illusion of control (Gainsbury, Philander, & Blaszczynski, Gainsbury et al., 2020a, b, c). This account is supported by the results of a self-report study using participants from US states where SGMs are currently legal (Gainsbury, Philander, & Grattan, Gainsbury et al., 2020b). That study found that, in comparison to those who had only used EGMs, gamblers who had used SGMs had: higher rates of disordered gambling severity, less objective knowledge of how EGMs work (according to an ad hoc but face valid set of 4 questions), and ironically higher self-reported knowledge of how EGMs work (Gainsbury Philander & Grattan., 2020b). These results add support to the previous sections warning around how various SGM features may interplay with biases common amongst gamblers, and particularly common amongst disordered gamblers. Some other evidence suggests that intention to gamble on SGMs is predicted by both positive attitudes toward SGMs and perceived positive social norms (Gainsbury, Philander, & Grattan, Gainsbury et al., 2020a), which aligns with predictions of the Theory of Reasoned Action (Fishbein, 1980).

One remaining issue is the extent to which any potential harms arising from the unique features of SGMs could be mitigated by providing informative warning labels to gamblers. There is a large body of literature on this topic in traditional EGMs, suggesting that the provision of most information is unlikely to have much effect on gamblers’ behaviour (Ginley et al., 2017; Harrigan et al., 2017). However, it has been suggested that cost of play information can reduce gamblers’ behaviour on a simulated EGM when it is appropriately “framed” (Newall et al., 2022). The findings of an online self-report survey indicate that participants did not exhibit a difference in positive attitudes towards SGMs or overconfidence in their knowledge about SGMs, regardless of whether the machine was labelled as having outcomes determined by a mix of skill and chance or not (Philander & Gainsbury, 2020). This last result suggests that further work is needed to see if a more sophisticated warning label can modify relevant aspects of SGM attitudes and behaviour given the variability of designs in the current SGM market.

## Discussion and Conclusion

This literature review was devised with the purpose of providing a preliminary review of SGM features that either seek to mimic video gaming or that could contribute to harmful engagement. The review further considered whether SGMs might primarily attract a new group of people who generally do not gamble, and to consider which current gamblers might find SGMs appealing.

SGMs are an emerging gambling product which uniquely blurs the lines between unskilled and skilled gambling. The novelty of SGMs is one factor which makes it hard to predict their eventual association with disordered and harmful gambling. Another factor is the variability present within existing SGM designs, which may have been designed with quite different segments of consumers in mind, and possibly have differential effects across different types of gamblers. Additionally, SGMs’ similarities with EGMs, which have the strongest association with disordered/harmful gambling out of all established gambling products (Productivity Commission, 2010), is one reason to expect a potentially similar association with this new gambling product, which is a prediction supported by one of the only empirical studies performed on SGMs to date (Gainsbury et al., 2020b). In addition, SGMs can have a number of unique product features in comparison to traditional EGMs, as highlighted via the VICES framework (Armstrong et al., 2017), which may play on established vulnerabilities amongst disordered gamblers, especially the illusion of control (Clark & Wohl, 2021).

Finally, while SGMs are highly varied in content, and evidence on their popularity is scarce, it appears possible that at least some SGMs could help attract a new group of consumers who generally do not gamble due, for example, to their similarities with video games. Furthermore, whilst empirical evidence is still developing, and with the caveat that SGMs could have varied influences given their broad range of potential design, it appears that disordered gamblers are the group of current gamblers who are most likely to engage with SGMs (Gainsbury et al., 2020b).

In conclusion, this review aimed to cover key areas from gambling research that are relevant to SGMs, and to pose a number of questions which might be answered by future developments of SGM design and the relevant accumulating evidence base.
